# 1′-Benzylspiro­[chromene-2,4′-piperi­dine]-4-carbonitrile

**DOI:** 10.1107/S1600536812035568

**Published:** 2012-08-23

**Authors:** P. Rajalakshmi, N. Srinivasan, R. V. Krishnakumar

**Affiliations:** a Department of Physics, Thiagarajar College, Madurai 625 009, India

## Abstract

In the title compound, C_21_H_20_N_2_O, the piperidine ring adopts a chair conformation while the pyran ring adopts a screw-boat conformation. The piperidine ring forms dihedral angles of 65.75 (3) and 67.79 (5)° with the chroman and methyl-substituted benzene rings, respectively. The crystal structure features weak C—H⋯π and π–π [centroid–centroid distance = 3.8098 (8) Å] inter­actions.

## Related literature

For the biological activity of piperidine­carbonitrile derivatives, see: Cardellicchio *et al.* (2010 ▶); Huang *et al.* (2008 ▶); Kumar *et al.* (2010 ▶); Arbiser *et al.* (2007 ▶). For uses of piperidine­carbonitrile derivatives, see: Barth *et al.* (2005 ▶); Vicente (2001 ▶); Terasaki *et al.* (2003 ▶). For industrial applications, see: Eller *et al.* (2002 ▶). For puckering prameters, see: Cremer & Pople (1975 ▶).
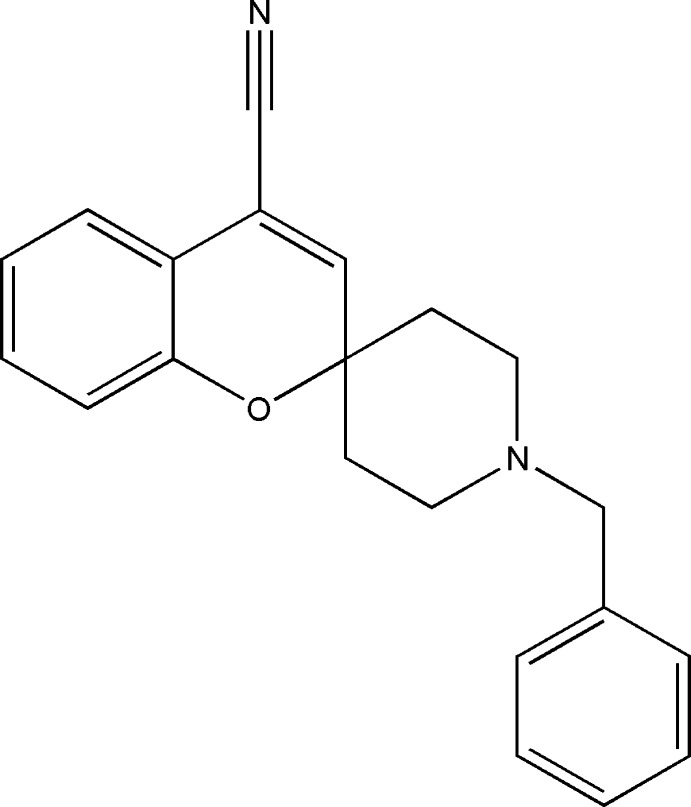


## Experimental

### 

#### Crystal data

C_21_H_20_N_2_O
*M* *_r_* = 316.39
Monoclinic, 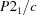

*a* = 15.1666 (9) Å
*b* = 10.0472 (6) Å
*c* = 12.4360 (8) Å
β = 113.931 (2)°
*V* = 1732.11 (18) Å^3^
*Z* = 4
Mo *K*α radiation
μ = 0.08 mm^−1^
*T* = 298 K
0.35 × 0.30 × 0.25 mm

#### Data collection

Bruker Kappa APEXII diffractometer
Absorption correction: multi-scan (*SADABS*; Sheldrick, 2008 ▶) *T*_min_ = 0.974, *T*_max_ = 0.981
24973 measured reflections
6238 independent reflections
3363 reflections with *I* > 2σ(*I*)
*R*_int_ = 0.033

#### Refinement

*R*[*F* ^2^ > 2σ(*F* ^2^)] = 0.049
*wR*(*F* ^2^) = 0.152
*S* = 1.01
6238 reflections
217 parameters
H-atom parameters constrained
Δρ_max_ = 0.17 e Å^−3^
Δρ_min_ = −0.17 e Å^−3^

### 

Data collection: *APEX2* (Bruker, 2004 ▶); cell refinement: *SAINT* (Bruker, 2004 ▶); data reduction: *SAINT*; program(s) used to solve structure: *SHELXS97* (Sheldrick, 2008 ▶); program(s) used to refine structure: *SHELXL97* (Sheldrick, 2008 ▶); molecular graphics: *PLUTON* (Spek, 2009) ▶; software used to prepare material for publication: *SHELXL97*.

## Supplementary Material

Crystal structure: contains datablock(s) I, global. DOI: 10.1107/S1600536812035568/gw2123sup1.cif

Structure factors: contains datablock(s) I. DOI: 10.1107/S1600536812035568/gw2123Isup2.hkl

Supplementary material file. DOI: 10.1107/S1600536812035568/gw2123Isup3.cml

Additional supplementary materials:  crystallographic information; 3D view; checkCIF report

## Figures and Tables

**Table 1:** Hydrogen-bond geometry (Å, °) *Cg*1 is the centroid of the C15–C20 ring.

*D*—H⋯*A*	*D*—H	H⋯*A*	*D*⋯*A*	*D*—H⋯*A*
C3—H3⋯*Cg*1^i^	0.93	2.95	3.7587 (15)	146

Symmetry code: (i) 
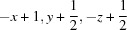
.

**Fig. 1.**
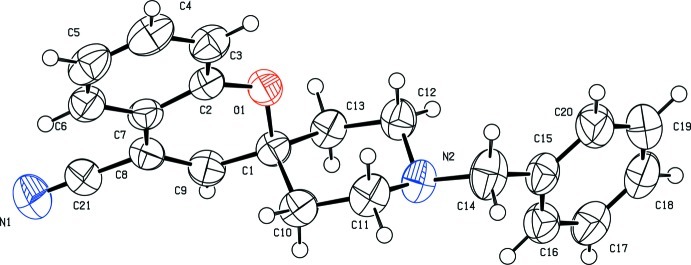
The molecular structure of the title compound showing 50% probability displacement ellipsoids.

**Fig. 2.**
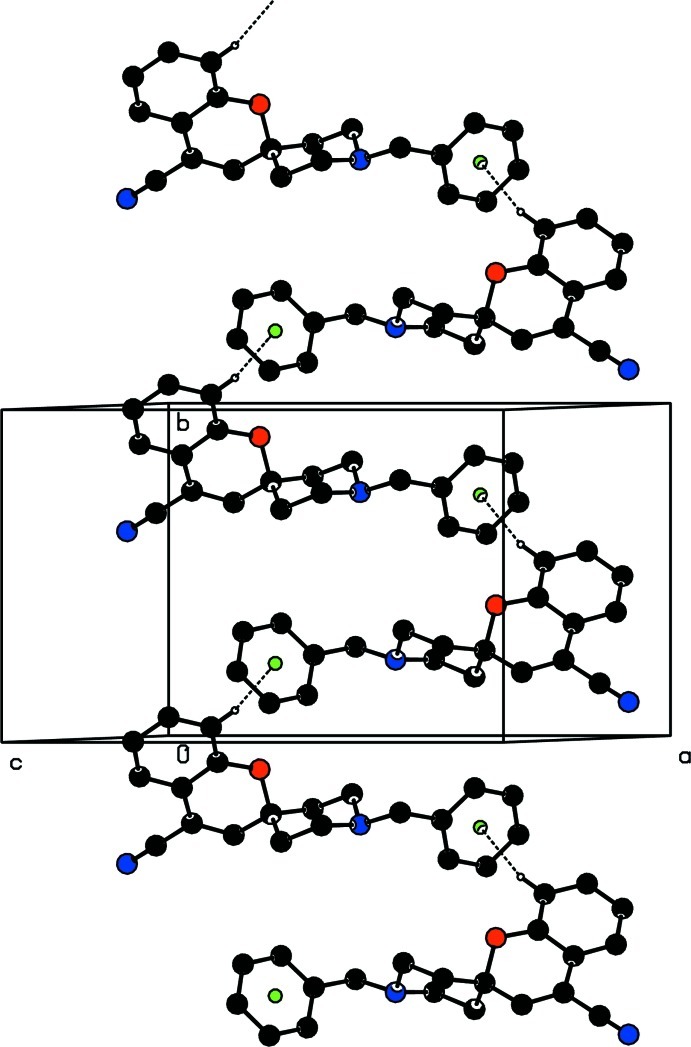
Part of the crystal structure of title compound showing the formation of a chain running along [010] direction generated by a C—H···π interaction. For the sake of clarity, the H atoms not involved in the motif have been omitted.

**Fig. 3.**
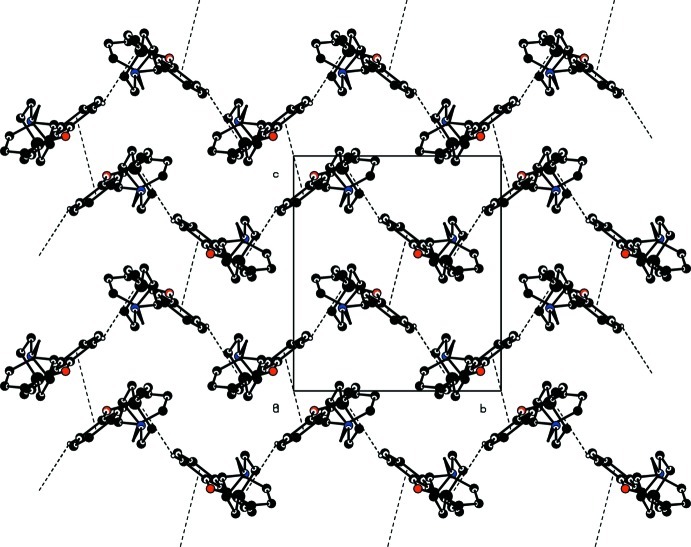
Crystal structure of title compound showing the formation of a extended sheet running along [011] plane generated by a C—H···π and π ···π interactions. For the sake of clarity, the H atoms not involved in the motif have been omitted

## supplementary crystallographic information

### Comment

Piperidine carbonitrile derivatives are used as sensitizers in photodynamic therapy (PDT) (Vicente, 2001) and in boron neutron capture therapy (BNCT) (Barth *et al.*, 2005) of brain tumors, which protects the central nervous system from drugs and endogenous molecules (Terasaki *et al.*, 2003) and exhibits good bioactivities (Cardellicchio *et al.* 2010; Huang *et al.* 2008; Kumar *et al.* 2010). Also, piperidines find application in the production of dipiperidinyl dithiuram tetrasulfide which is used as a rubber vulcanization accelerator (Eller *et al.* 2002). The piperidine structural motif is present in natural alkaloids of fire ant toxin solenopsin and is an inhibitor of phosphatidylinositol-3-kinase signalling and angiogenesis (Arbiser *et al.* 2007).

In the title molecule (Fig. 1), the puckering conformation (Cremer & Pople, 1975) of the pyran ring (C8/C7/C2/O1/C1/C9) is nearly screw boat (^5^S_4_) with parameters: Q = 0.3407 (12) Å, θ = 116.2 (2)° and φ = 213.0 (2)°. The deviation of O1 and C1 from the mean plane defined by the rest of the atoms is -0.6934 Å and 0.6178 Å, respectively. The puckering of the piperidine ring (N2/C10/C11/C1/C12/C13) with parameters of Q = 0.5660 (14) Å, θ = 173.13 (13) ° and φ = 181.5 (12) ° is close to ideal chair (^1^C_4_) and the deviations of N2 and C1 from the mean plane defined by the rest of the atoms by -0.6934 (16) Å and 0.6178 (17) Å, respectively.

The crystal structure of the title compound demonstrates the importance of weak interactions in optimizing the molecular aggregation in crystals. With the lone acceptor oxygen O1 unavailable for participation in intermolecular interactions for sterical reasons, the weak C–H···π and π···π interactions assume significance. A C3—H3···C*g*1 (1 - *x*, 1/2 + *y*, 1/2 - *z*), *Cg*1 being the centroid of the benzene ring defined by C15 – C20, having a distance of 2.95 Å and angle of 146°, generates chains running along the *b* axis. A C*g2*··· C*g*2 (-*x* + 1, *y* + 1/2, -*z* + 1/2) interaction, C*g*2 being the centroid of the benzene ring defined by C2 – C7, observed between two benzene rings of the chroman. The corresponding ring-centroid separation is 3.8098 (8) Å, with an interplanar spacing of *ca* 3.51 Å and a ring offset of *ca* 1.48 Å. These interactions generate a π-stacked extended sheets running parallel to the [011] direction (Fig. 3).

The accurate description of the crystal structure of title compound is of interest due to the absence of conventional hydrogen bonding and thus gains importance in the context of crystal structure prediction. Precise single-crystal X-ray investigations on similar compounds might throw light on the delicate nature of intermolecular interactions.

### Experimental

Trimethylsilylcyanide (1.2 mmol) was added to a mixture of 1'1'-benzyl-3, 4-dihydrospiro [1-benzopyran-2, 4'- piperidine]-4-one (1.0 mmol) and catalytic amount of ZnI_2_ in dichloromethane (10 vol), under a nitrogen atmosphere. The reaction mixture was stirred at 50°C for 6 h and then cooled to room temperature, dilute HCl (5 ml) was added and stirring continued for additional 2 h. The solution was extracted with ethylacetate (20 ml), dried over Na_2_SO_4_ and evaporated to dryness. The crude product was dissolved in benzene (10 ml), to which tosic acid (0.1 mmol) had been added and the solution was heated to reflux for 2 h. After completion of the reaction as indicated by *TLC*, the reaction mixture was concentrated under reduced pressure. The residue was diluted with ethylacetate (20 ml), washed with bicarbonate solution (10 ml) dried and concentrated. The crude product was purified by column chromatography to provide the desired product as colorless solid. Crystals of the title compound were grown from its solution in ethanol by slow evaporation at room temperature.

### Crystal data

**Table:**

C_21_H_20_N_2_O	*F*(000) = 672
*M**_r_* = 316.39	*D*_x_ = 1.213 Mg m^−^^3^
Monoclinic, *P*2_1_/*c*	Mo *K*α radiation, λ = 0.71073 Å
Hall symbol: -P 2ybc	Cell parameters from 6238 reflections
*a* = 15.1666 (9) Å	θ = 2.7–29.4°
*b* = 10.0472 (6) Å	µ = 0.08 mm^−^^1^
*c* = 12.4360 (8) Å	*T* = 298 K
β = 113.931 (2)°	Block, colourless
*V* = 1732.11 (18) Å^3^	0.35 × 0.30 × 0.25 mm
*Z* = 4	

### Data collection

**Table:**

Bruker Kappa APEXII diffractometer	6238 independent reflections
Radiation source: fine-focus sealed tube	3363 reflections with *I* > 2σ(*I*)
Graphite monochromator	*R*_int_ = 0.033
φ and ω scans	θ_max_ = 32.8°, θ_min_ = 2.5°
Absorption correction: multi-scan (*SADABS*; Sheldrick, 2008)	*h* = −23→22
*T*_min_ = 0.974, *T*_max_ = 0.981	*k* = −15→15
24973 measured reflections	*l* = −18→17

### Refinement

**Table:**

Refinement on *F*^2^	Primary atom site location: structure-invariant direct methods
Least-squares matrix: full	Secondary atom site location: difference Fourier map
*R*[*F*^2^ > 2σ(*F*^2^)] = 0.049	Hydrogen site location: inferred from neighbouring sites
*wR*(*F*^2^) = 0.152	H-atom parameters constrained
*S* = 1.01	*w* = 1/[σ^2^(*F*_o_^2^) + (0.0684*P*)^2^ + 0.085*P*] where *P* = (*F*_o_^2^ + 2*F*_c_^2^)/3
6238 reflections	(Δ/σ)_max_ < 0.001
217 parameters	Δρ_max_ = 0.17 e Å^−^^3^
0 restraints	Δρ_min_ = −0.17 e Å^−^^3^

### Special details

**Table:**

Geometry. All e.s.d.'s (except the e.s.d. in the dihedral angle between two l.s. planes) are estimated using the full covariance matrix. The cell e.s.d.'s are taken into account individually in the estimation of e.s.d.'s in distances, angles and torsion angles; correlations between e.s.d.'s in cell parameters are only used when they are defined by crystal symmetry. An approximate (isotropic) treatment of cell e.s.d.'s is used for estimating e.s.d.'s involving l.s. planes.
Refinement. Refinement of *F*^2^ against ALL reflections. The weighted *R*-factor *wR* and goodness of fit *S* are based on *F*^2^, conventional *R*-factors *R* are based on *F*, with *F* set to zero for negative *F*^2^. The threshold expression of *F*^2^ > σ(*F*^2^) is used only for calculating *R*-factors(gt) *etc*. and is not relevant to the choice of reflections for refinement. *R*-factors based on *F*^2^ are statistically about twice as large as those based on *F*, and *R*- factors based on ALL data will be even larger.

### Fractional atomic coordinates and isotropic or equivalent isotropic displacement parameters (Å^2^)

**Table:**

	*x*	*y*	*z*	*U*_iso_*/*U*_eq_	
O1	0.20929 (5)	0.90059 (8)	0.08396 (8)	0.0525 (2)	
N1	−0.11382 (9)	0.61311 (13)	−0.09299 (14)	0.0841 (4)	
N2	0.43051 (7)	0.73556 (11)	0.14770 (9)	0.0543 (3)	
C1	0.22110 (8)	0.76526 (11)	0.05117 (10)	0.0458 (3)	
C2	0.13926 (8)	0.92304 (11)	0.12528 (10)	0.0460 (3)	
C3	0.15085 (9)	1.02973 (13)	0.19913 (11)	0.0579 (3)	
H3	0.2068	1.0808	0.2243	0.070*	
C4	0.07830 (11)	1.06010 (15)	0.23549 (12)	0.0686 (4)	
H4	0.0859	1.1318	0.2858	0.082*	
C5	−0.00460 (10)	0.98619 (15)	0.19857 (12)	0.0675 (4)	
H5	−0.0529	1.0080	0.2235	0.081*	
C6	−0.01627 (9)	0.87965 (13)	0.12457 (12)	0.0578 (3)	
H6	−0.0727	0.8297	0.0996	0.069*	
C7	0.05550 (8)	0.84568 (11)	0.08656 (10)	0.0460 (3)	
C8	0.04828 (8)	0.73709 (11)	0.00511 (11)	0.0495 (3)	
C9	0.12497 (8)	0.70138 (12)	−0.01467 (11)	0.0516 (3)	
H9	0.1187	0.6359	−0.0701	0.062*	
C10	0.27778 (9)	0.68420 (13)	0.16113 (11)	0.0540 (3)	
H10A	0.2448	0.6875	0.2135	0.065*	
H10B	0.2797	0.5920	0.1391	0.065*	
C11	0.37980 (9)	0.73457 (15)	0.22566 (11)	0.0600 (3)	
H11A	0.3784	0.8239	0.2544	0.072*	
H11B	0.4140	0.6778	0.2929	0.072*	
C12	0.38213 (8)	0.82572 (14)	0.04959 (11)	0.0562 (3)	
H12A	0.4177	0.8293	0.0001	0.067*	
H12B	0.3809	0.9146	0.0794	0.067*	
C13	0.27973 (8)	0.77961 (13)	−0.02271 (10)	0.0515 (3)	
H13A	0.2816	0.6946	−0.0587	0.062*	
H13B	0.2482	0.8432	−0.0853	0.062*	
C14	0.53183 (9)	0.77325 (17)	0.21193 (13)	0.0705 (4)	
H14A	0.5590	0.7223	0.2843	0.085*	
H14B	0.5352	0.8667	0.2329	0.085*	
C15	0.59135 (8)	0.75002 (13)	0.14180 (12)	0.0566 (3)	
C16	0.58601 (8)	0.63042 (14)	0.08502 (12)	0.0605 (3)	
H16	0.5452	0.5641	0.0903	0.073*	
C17	0.63989 (9)	0.60737 (15)	0.02078 (13)	0.0671 (4)	
H17	0.6346	0.5264	−0.0176	0.081*	
C18	0.70137 (9)	0.70297 (17)	0.01293 (14)	0.0722 (4)	
H18	0.7380	0.6872	−0.0303	0.087*	
C19	0.70834 (10)	0.82101 (18)	0.06891 (18)	0.0856 (5)	
H19	0.7501	0.8862	0.0640	0.103*	
C20	0.65394 (10)	0.84503 (15)	0.13306 (16)	0.0792 (5)	
H20	0.6595	0.9264	0.1709	0.095*	
C21	−0.04213 (9)	0.66841 (13)	−0.05118 (13)	0.0603 (3)	

### Atomic displacement parameters (Å^2^)

**Table:**

	*U*^11^	*U*^22^	*U*^33^	*U*^12^	*U*^13^	*U*^23^
O1	0.0519 (4)	0.0463 (5)	0.0652 (6)	−0.0025 (3)	0.0299 (4)	−0.0075 (4)
N1	0.0619 (7)	0.0730 (8)	0.1129 (11)	−0.0153 (6)	0.0308 (7)	−0.0042 (8)
N2	0.0463 (5)	0.0737 (7)	0.0408 (6)	0.0046 (4)	0.0154 (4)	−0.0039 (5)
C1	0.0492 (5)	0.0463 (6)	0.0452 (6)	0.0024 (4)	0.0225 (5)	−0.0040 (5)
C2	0.0491 (5)	0.0444 (6)	0.0453 (6)	0.0069 (5)	0.0200 (5)	0.0035 (5)
C3	0.0613 (7)	0.0534 (7)	0.0541 (8)	0.0061 (6)	0.0183 (6)	−0.0061 (6)
C4	0.0829 (9)	0.0674 (9)	0.0556 (8)	0.0221 (7)	0.0281 (7)	−0.0051 (7)
C5	0.0751 (9)	0.0775 (9)	0.0617 (9)	0.0274 (7)	0.0399 (7)	0.0133 (7)
C6	0.0576 (6)	0.0607 (8)	0.0627 (8)	0.0106 (6)	0.0323 (6)	0.0167 (6)
C7	0.0500 (5)	0.0427 (6)	0.0473 (7)	0.0063 (4)	0.0219 (5)	0.0094 (5)
C8	0.0505 (6)	0.0436 (6)	0.0535 (7)	−0.0026 (5)	0.0203 (5)	0.0046 (5)
C9	0.0550 (6)	0.0467 (6)	0.0524 (7)	−0.0021 (5)	0.0211 (5)	−0.0069 (5)
C10	0.0611 (7)	0.0600 (7)	0.0474 (7)	0.0074 (5)	0.0286 (6)	0.0056 (6)
C11	0.0629 (7)	0.0780 (9)	0.0389 (7)	0.0091 (6)	0.0203 (6)	0.0025 (6)
C12	0.0528 (6)	0.0717 (8)	0.0490 (7)	0.0022 (6)	0.0257 (5)	0.0028 (6)
C13	0.0517 (6)	0.0646 (7)	0.0395 (6)	0.0074 (5)	0.0197 (5)	0.0039 (5)
C14	0.0525 (7)	0.0940 (11)	0.0560 (8)	−0.0006 (7)	0.0128 (6)	−0.0216 (8)
C15	0.0404 (5)	0.0647 (8)	0.0545 (8)	0.0012 (5)	0.0086 (5)	−0.0105 (6)
C16	0.0505 (6)	0.0618 (8)	0.0643 (9)	−0.0030 (6)	0.0181 (6)	−0.0072 (6)
C17	0.0575 (7)	0.0708 (9)	0.0673 (9)	0.0113 (6)	0.0196 (6)	−0.0095 (7)
C18	0.0485 (7)	0.0940 (11)	0.0735 (10)	0.0155 (7)	0.0240 (7)	0.0089 (8)
C19	0.0569 (8)	0.0800 (11)	0.1225 (15)	−0.0040 (7)	0.0391 (9)	0.0061 (10)
C20	0.0590 (7)	0.0624 (9)	0.1117 (13)	−0.0068 (6)	0.0298 (8)	−0.0195 (8)
C21	0.0557 (7)	0.0515 (7)	0.0740 (9)	−0.0034 (5)	0.0266 (6)	0.0010 (6)

### Geometric parameters (Å, º)

**Table:**

O1—C2	1.3732 (12)	C10—H10A	0.9700
O1—C1	1.4512 (13)	C10—H10B	0.9700
N1—C21	1.1413 (16)	C11—H11A	0.9700
N2—C12	1.4565 (16)	C11—H11B	0.9700
N2—C11	1.4618 (14)	C12—C13	1.5173 (17)
N2—C14	1.4652 (16)	C12—H12A	0.9700
C1—C9	1.4965 (16)	C12—H12B	0.9700
C1—C13	1.5227 (14)	C13—H13A	0.9700
C1—C10	1.5227 (17)	C13—H13B	0.9700
C2—C3	1.3757 (16)	C14—C15	1.5063 (17)
C2—C7	1.3977 (16)	C14—H14A	0.9700
C3—C4	1.3827 (18)	C14—H14B	0.9700
C3—H3	0.9300	C15—C16	1.3793 (18)
C4—C5	1.369 (2)	C15—C20	1.3812 (19)
C4—H4	0.9300	C16—C17	1.3746 (18)
C5—C6	1.375 (2)	C16—H16	0.9300
C5—H5	0.9300	C17—C18	1.369 (2)
C6—C7	1.3936 (15)	C17—H17	0.9300
C6—H6	0.9300	C18—C19	1.357 (2)
C7—C8	1.4623 (16)	C18—H18	0.9300
C8—C9	1.3319 (15)	C19—C20	1.382 (2)
C8—C21	1.4380 (17)	C19—H19	0.9300
C9—H9	0.9300	C20—H20	0.9300
C10—C11	1.5122 (18)		
			
C2—O1—C1	117.47 (8)	C10—C11—H11A	109.5
C12—N2—C11	109.76 (9)	N2—C11—H11B	109.5
C12—N2—C14	110.89 (11)	C10—C11—H11B	109.5
C11—N2—C14	110.97 (10)	H11A—C11—H11B	108.1
O1—C1—C9	110.59 (8)	N2—C12—C13	110.69 (10)
O1—C1—C13	104.46 (9)	N2—C12—H12A	109.5
C9—C1—C13	112.85 (10)	C13—C12—H12A	109.5
O1—C1—C10	109.75 (9)	N2—C12—H12B	109.5
C9—C1—C10	109.38 (9)	C13—C12—H12B	109.5
C13—C1—C10	109.72 (9)	H12A—C12—H12B	108.1
O1—C2—C3	117.97 (10)	C12—C13—C1	112.29 (9)
O1—C2—C7	120.84 (10)	C12—C13—H13A	109.1
C3—C2—C7	121.02 (10)	C1—C13—H13A	109.1
C2—C3—C4	119.16 (12)	C12—C13—H13B	109.1
C2—C3—H3	120.4	C1—C13—H13B	109.1
C4—C3—H3	120.4	H13A—C13—H13B	107.9
C5—C4—C3	120.99 (13)	N2—C14—C15	112.75 (10)
C5—C4—H4	119.5	N2—C14—H14A	109.0
C3—C4—H4	119.5	C15—C14—H14A	109.0
C4—C5—C6	119.86 (11)	N2—C14—H14B	109.0
C4—C5—H5	120.1	C15—C14—H14B	109.0
C6—C5—H5	120.1	H14A—C14—H14B	107.8
C5—C6—C7	120.75 (12)	C16—C15—C20	117.47 (12)
C5—C6—H6	119.6	C16—C15—C14	120.45 (12)
C7—C6—H6	119.6	C20—C15—C14	122.07 (13)
C6—C7—C2	118.22 (11)	C17—C16—C15	121.26 (13)
C6—C7—C8	124.64 (11)	C17—C16—H16	119.4
C2—C7—C8	117.11 (9)	C15—C16—H16	119.4
C9—C8—C21	120.83 (11)	C18—C17—C16	120.38 (14)
C9—C8—C7	120.22 (10)	C18—C17—H17	119.8
C21—C8—C7	118.94 (10)	C16—C17—H17	119.8
C8—C9—C1	120.88 (11)	C19—C18—C17	119.33 (13)
C8—C9—H9	119.6	C19—C18—H18	120.3
C1—C9—H9	119.6	C17—C18—H18	120.3
C11—C10—C1	112.40 (10)	C18—C19—C20	120.53 (14)
C11—C10—H10A	109.1	C18—C19—H19	119.7
C1—C10—H10A	109.1	C20—C19—H19	119.7
C11—C10—H10B	109.1	C15—C20—C19	121.00 (14)
C1—C10—H10B	109.1	C15—C20—H20	119.5
H10A—C10—H10B	107.9	C19—C20—H20	119.5
N2—C11—C10	110.53 (10)	N1—C21—C8	178.19 (16)
N2—C11—H11A	109.5		
			
C2—O1—C1—C9	42.04 (13)	C9—C1—C10—C11	174.04 (9)
C2—O1—C1—C13	163.72 (9)	C13—C1—C10—C11	49.76 (13)
C2—O1—C1—C10	−78.72 (11)	C12—N2—C11—C10	61.96 (14)
C1—O1—C2—C3	154.00 (11)	C14—N2—C11—C10	−175.13 (11)
C1—O1—C2—C7	−30.59 (14)	C1—C10—C11—N2	−56.68 (14)
O1—C2—C3—C4	175.57 (11)	C11—N2—C12—C13	−61.80 (13)
C7—C2—C3—C4	0.17 (18)	C14—N2—C12—C13	175.24 (9)
C2—C3—C4—C5	−0.4 (2)	N2—C12—C13—C1	56.38 (13)
C3—C4—C5—C6	0.3 (2)	O1—C1—C13—C12	68.12 (12)
C4—C5—C6—C7	0.06 (19)	C9—C1—C13—C12	−171.70 (10)
C5—C6—C7—C2	−0.26 (17)	C10—C1—C13—C12	−49.46 (13)
C5—C6—C7—C8	−177.93 (11)	C12—N2—C14—C15	−68.92 (15)
O1—C2—C7—C6	−175.12 (10)	C11—N2—C14—C15	168.82 (12)
C3—C2—C7—C6	0.15 (17)	N2—C14—C15—C16	−48.09 (18)
O1—C2—C7—C8	2.72 (15)	N2—C14—C15—C20	132.97 (15)
C3—C2—C7—C8	177.99 (11)	C20—C15—C16—C17	−0.9 (2)
C6—C7—C8—C9	−171.55 (12)	C14—C15—C16—C17	−179.90 (12)
C2—C7—C8—C9	10.76 (17)	C15—C16—C17—C18	0.8 (2)
C6—C7—C8—C21	7.31 (18)	C16—C17—C18—C19	−0.2 (2)
C2—C7—C8—C21	−170.39 (11)	C17—C18—C19—C20	−0.1 (2)
C21—C8—C9—C1	−174.98 (11)	C16—C15—C20—C19	0.5 (2)
C7—C8—C9—C1	3.85 (18)	C14—C15—C20—C19	179.50 (14)
O1—C1—C9—C8	−29.20 (16)	C18—C19—C20—C15	0.0 (3)
C13—C1—C9—C8	−145.79 (12)	C9—C8—C21—N1	122 (5)
C10—C1—C9—C8	91.78 (13)	C7—C8—C21—N1	−57 (5)
O1—C1—C10—C11	−64.47 (12)		

### Hydrogen-bond geometry (Å, º)

Cg1 is the centroid of the C15–C20 ring.

**Table:**

*D*—H···*A*	*D*—H	H···*A*	*D*···*A*	*D*—H···*A*
C3—H3···*Cg*1^i^	0.93	2.95	3.7587 (15)	146

Symmetry code: (i) −*x*+1, *y*+1/2, −*z*+1/2.
